# Risk prediction of advanced colorectal neoplasia varies by race and neighbourhood socioeconomic status

**DOI:** 10.1136/fmch-2024-002892

**Published:** 2024-05-30

**Authors:** Xiangqing Sun, Zhengyi Chen, Gregory S Cooper, Nathan A Berger, Claudia Coulton, Li Li

**Affiliations:** 1 Department of Family Medicine, University of Virginia, Charlottesville, Virginia, USA; 2 Department of Population and Quantitative Health Sciences, Case Western Reserve University, Cleveland, Ohio, USA; 3 University Hospitals Cleveland Medical Center, Cleveland, Ohio, USA; 4 Case Comprehensive Cancer Center, Cleveland, Ohio, USA; 5 Jack, Joseph and Morton Mandel School of Applied Social Sciences, Case Western Reserve University, Cleveland, Ohio, USA

**Keywords:** Epidemiology, Environment and Public Health, Public Health

## Abstract

**Objective:**

Neighbourhood deprivation increases the risk of colorectal neoplasia and contributes to racial disparities observed in this disease. Developing race-specific advanced colorectal neoplasia (ACN) prediction models that include neighbourhood socioeconomic status has the potential to improve the accuracy of prediction.

**Methods:**

The study includes 1457 European Americans (EAs) and 936 African Americans (AAs) aged 50–80 years undergoing screening colonoscopy. Race-specific ACN risk prediction models were developed for EAs and AAs, respectively. Area Deprivation Index (ADI), derived from 17 variables of neighbourhood socioeconomic status, was evaluated by adding it to the ACN risk prediction models. Prediction accuracy was evaluated by concordance statistic (C-statistic) for discrimination and Hosmer-Lemeshow goodness-of-fit test for calibration.

**Results:**

With fewer predictors, the EA-specific and AA-specific prediction models had better prediction accuracy in the corresponding race/ethnic subpopulation than the overall model. Compared with the overall model which had poor calibration (*P*_Calibration_=0.053 in the whole population and *P*_Calibration_=0.011 in AAs), the EA model had C-statistic of 0.655 (95% CI 0.594 to 0.717) and *P*_Calibration_=0.663; and the AA model had C-statistic of 0.637 ((95% CI 0.572 to 0.702) and *P*_Calibration_=0.810. ADI was a significant predictor of ACN in EAs (OR=1.24 ((95% CI 1.03 to 1.50), *P*=0.029), but not in AAs (OR=1.07 ((95% CI 0.89 to 1.28), *P*=0.487). Adding ADI to the EA-specific ACN prediction model substantially improved ACN calibration accuracy of the prediction across area deprivation groups (*P*_Calibration_=0.924 with ADI vs *P*_Calibration_=0.140 without ADI) in EAs.

**Conclusions:**

Neighbourhood socioeconomic status is an important factor to consider in ACN risk prediction modeling. Moreover, non-race-specific prediction models have poor generalisability. Race-specific prediction models incorporating neighbourhood socioeconomic factors are needed to improve ACN prediction accuracy.

**WHAT IS ALREADY KNOWN ON THIS TOPIC**

- Neighbourhood socioeconomic status (nSES) has been increasingly associated with the development of advanced colorectal neoplasia (ACN). While nSES has been shown to improve the accuracy of cardiovascular risk prediction, no previous studies have included this factor in ACN risk prediction modelling.

**WHAT THIS STUDY ADDS**

- Our study reveals that incorporating patients’ nSES improves the calibration accuracy of ACN risk prediction across area deprivation groups in European Americans. Failure to consider nSES led to overestimation of ACN risk for people living in affluent neighbourhood and underestimation of ACN risk for those living in disadvantaged neighbourhood. Moreover, there is no one-fit-all prediction model for all racial groups, and race-specific models are needed for accurate ACN risk prediction.

**HOW THIS STUDY MIGHT AFFECT RESEARCH, PRACTICE OR POLICY**

- Our findings underscore the importance of considering nSES in ACN risk prediction modelling. It may guide future research and potentially influence targeted prevention policies for colorectal neoplasia.

## Introduction

Colorectal cancer (CRC) is preventable through screening for early detection of cancer or removal of precursor lesions, such as advanced colorectal adenoma.^1 2^ Other than individual-level behavioural and lifestyle risk factors, neighbourhood socioeconomic status (nSES) has been associated with CRC risk.^3 4^ Studies have found that residential place had direct and indirect influences on health outcomes.^5^ CRC risk has clustered in areas with concentrated social disadvantage.^6^ For instance, individuals living in the lower Mississippi delta hotspot have a 40% higher risk of CRC death than in non-hotspot areas of the USA.^7^ Low SES neighbourhoods with high poverty rates and low education levels had higher risk of developing CRC even after accounting for other known risk factors,^8^ and the association was strongest in the rectum.^9^ Moreover, low nSES was associated with advanced stage CRC^10^ and higher risk of CRC mortality.^11^ In a study by Dalton *et al*,^12^ nSES was found to improve accuracy of cardiovascular risk prediction. Integrating nSES in the risk prediction model has the potential to improve the prediction accuracy and help reduce CRC disparities.

Advanced colorectal neoplasia (ACN) includes advanced, precancerous adenoma and CRC. It is a desirable target for CRC screening, early detection and surveillance.^13^ African Americans (AAs) have higher incidence and mortality rates and shorter survival for CRC than European Americans (EAs)^2 14^ and develop CRC at younger age.^15^ Moreover, advanced proximal colon cancers, which are associated with a worse prognosis compared with distal cancers,^16^ occur more frequently in AAs than in EAs.^17^ The observed disparities in CRC risk between AAs and EAs are linked to differences in environmental exposures, physical exercise, dietary, nutritional factors, access to healthcare and tumour molecular mechanisms.^15 18^ A number of risk prediction model for ACN have been developed to identify people at increased risk.^19–24^ These models are largely based on white or Asian populations^19–21 23 24^ or a population of diverse ethnicity.^22^ None of them has considered nSES for model development.

In this study, we aim to evaluate the association of nSES with ACN risk and assess its impact on ACN prediction. To address racial differences of ACN risk between EAs and AAs, we developed race-specific ACN prediction models for the two race groups, respectively, and evaluated the impact of nSES in the race-specific prediction models. Our study is the first to address nSES in ACN risk prediction and to develop race-specific ACN risk prediction models in AAs.

## Materials and methods

### Study population

Patients undergoing routine colonoscopy were recruited between 2006 and 2018 from University Hospitals Cleveland Medical Center and affiliated gastroenterology practices in the surrounding areas in Cleveland, Ohio. The detail of the cohort was described elsewhere.^25^ Briefly, potential participants are asymptomatic patients who were referred to the practices for CRC screening. They were 50 years and older and had no colonoscopy exam within the last 10 years or had a positive family history of CRC that met the American Cancer Society CRC screening recommendation to undergo screening colonoscopy at a younger age. Patients were at least 30 years old, did not have inflammatory bowel diseases (such as Crohn’s disease or ulcerative colitis) or had been diagnosed with CRC or polyps or any form of cancer. We collected demographics, behavioural and lifestyle risk factors through phone interview and validated questionnaires as described before.^25^

In this study, we only included colonoscopy screening patients who were aged 50–80 years old (n=2393) and were self-reported as either EAs or AAs. We excluded those who were younger than 50 years old. We also excluded patients of other races (n=78) because only ~3% of patients were in this category and only two of them had screening-detected ACN.

Patients were classified as ACN cases (n=171) if their colonoscopy pathology reports confirmed the diagnosis of adenocarcinoma of the colon or rectum, or advanced adenoma (a tubular adenoma or sessile serrated adenoma or polyp ≥10 mm in size, or an adenomatous polyp with villous histology or high-grade dysplasia.^26^ All others were considered as controls (n=2222) in our analysis.

### Statistical analyses

#### Univariate association

For binary variables, significance of univariate association of each demographic and behavioural risk factors with ACN risk was evaluated by χ^2^ test. For continuous variables, univariate association was evaluated by t-test assuming unequal variances in ACN cases and controls. OR was evaluated by univariate logistic regression.

#### ACN risk prediction models

##### Model development

We developed ACN risk prediction models for the EAs, the AAs and both populations combined, respectively. In each model, risk factors with univariate association with ACN (*P*<0.10) were included as candidate predictors specific to the corresponding population. Sex, body mass index (BMI), alcohol drinking, smoking, family history of CRC in first-degree relatives and red meat intake have been identified as risk factors for CRC.^20 22 27 28^ These variables were included as candidate predictors in developing prediction models, regardless of the significance level of their univariate associations in the corresponding population.

We next fitted multivariate logistic regression models to predict risk of ACN. We conducted a bootstrap sampling model selection procedure to obtain a stable and robust prediction model.^22 29^ We first fitted a model that included all candidate predictors and employed fast backward selection to eliminate predictors with *P* values >0.10 based on an approximate Wald test for predictors in the logistic regression model.^30^ The model selection was repeated 1000 times by bootstrap sampling. In each selection procedure, we did a random bootstrap sampling (with replacement) of the same number of subjects from the dataset and then performed the model fitting and backward selection with the sampled data. This procedure was repeated 1000 times. The final predictors were selected according to their frequency of being selected in the 1000 best-fitting models: we selected those with selection frequencies >0.4 and/or above a big frequency drop in the plot of ordered selection frequencies (online supplemental figure 1).

10.1136/fmch-2024-002892.supp1 Supplementary data

We further checked the redundancy of the selected predictors of a prediction model by fitting least absolute shrinkage and selection operator (LASSO) regression^31^ with the selected predictors. LASSO regression performs both variable selection and shrinkage to statistical models. It filters out the predictors that have minimal impact on the model (ie, the estimates of standardised variables close to 0). In LASSO regression, we performed a threefold cross-validation based on the prediction accuracy of area under the curve criterion for the model selection. No predictor was further eliminated by LASSO.

##### Model evaluation

Two assessments, the calibration accuracy and the discrimination accuracy, were used to evaluate the prediction accuracy of the prediction models. Calibration accuracy measures how well the average predicted risk probabilities agree with observed disease rates across all subgroups of the population, and it was evaluated by Hosmer-Lemeshow goodness of fit test.^32^ Discrimination accuracy measures how well the prediction model can separate affected from unaffected subjects, and it was evaluated by C-statistic (area under the receiver-operating curve), and its 95% CI was produced by applying the prediction model to 2000 imputed bootstrap datasets.

#### Evaluating Area Deprivation Index in ACN prediction

nSES was measured using the Area Deprivation Index (ADI).^33^ It is calculated based on 17 variables at the census tract level from the American Community Survey 5-year estimates (2013–2017). These variables include indicators of poverty, education, housing and employment (online supplemental table 1). The 17 variables were transformed into their principal components, and the first principal component, which accounts for the largest amount of the overall variability, is used as the ADI.^12^ ADI is scaled to be between 0 and 100.^34^

In order to study if adding ADI improves the ACN prediction compared with the model without ADI, we fitted a new prediction model with two predictors: ADI and the predictions (predicted risk values) from the model without ADI. There are 598 census tract-level communities in this study, and individuals are nested in census tract-level neighbourhoods. In order to account for the correlation within census tract, we fitted a mixed effect logistic regression model with the two predictors and an additional term of random effects for census tracts, assuming that there is variation of baseline risk across census tracts.^35^ The model estimates were based on an adaptive Gaussian Hermite approximation of the likelihood,^36^ and we used 10 integration points to obtain more accurate convergence to the maximum likelihood estimates. ADI as a predictor was evaluated in the three prediction models by the likelihood ratio test, and the model prediction accuracy with ADI was evaluated by discrimination accuracy (C-statistic) and calibration accuracy across area deprivation groups.

To evaluate how much ADI accounts for the local variation of ACN risk after including ADI in prediction, we first fit the null model, which is a mixed model with two elements: the fixed effect of predicted risks from the model without ADI, and the random effects of census tracts. We then fit the full model that adds ADI as an additional fixed effect, which is the aforementioned mixed effect ACN prediction model. We compared the variance from the random effects (ie, census tracts) of the two models, denoted as Var_Null_ and Var_Full,_ respectively, and the proportion of census-tract level variation of ACN risk that is accounted for by ADI is calculated as: (Var_Null_ – Var_Full_)/Var_Null_.

## Results

### Patient characteristics

As shown in online supplemental figure 2, we included 2393 individuals aged between 50 and 80 years. Of these, 1457 (60.9%) are EAs and 936 (39.1%) are AAs. The characteristics of the study population and risk factors are presented in table 1.

**Table 1:** Demographic and clinical characteristics of the study population

Characteristic	Overall	European Americans	African Americans
Number of patients	2393	1457	936
Sex, n (%)			
Male	934 (39.0)	631 (43.3)	303 (32.4)
Female	1459 (61.0)	826 (56.7)	633 (67.6)
Race, n (%)			
European American	1457 (60.9)		
African American	936 (39.1)		
Age, years, mean (SD)	57.8 (7.2)	57.8 (7.0)	57.9 (7.4)
BMI, kg/m^2^, mean (SD)	29.6 (7.0)	28.3 (5.9)	31.8 (7.9)
Height, cm, mean (SD)	169.2 (9.9)	170.2 (9.6)	167.5 (10.2)
Waist–hip ratio (SD)	9.2 (0.9)	9.0 (0.9)	9.4 (0.9)
ACN (%)	171 (7.1)	92 (6.3)	79 (8.4)
History of CRC in first-degree relatives (%)	255 (10.9)	154 (10.7)	101 (11.2)
History of CRC in all relatives (%)	523 (22.7)	346 (24.4)	177 (20.0)
Diabetes (%)	356 (15.3)	131 (9.1)	225 (25.0)
Physical activities, metabolic equivalents, mean (SD)	3.3 (2.6)	3.7 (2.8)	2.6 (2.3)
Frequencies of red meat per week, mean (SD)	3.1 (3.2)	2.9 (2.8)	3.3 (3.8)
Frequencies of alcohol per week, (SD)	3.0 (9.1)	3.0 (5.6)	3.0 (12.9)
NSAIDs (%)	772 (33.0)	509 (35.3)	263 (29.2)
Calcium (%)	471 (20.1)	371 (25.8)	100 (11.1)
Aspirin (%)	636 (27.2)	408 (28.3)	228 (25.3)
Ibuprophen (%)	227 (9.9)	167 (11.9)	60 (6.8)
Years of smoking (SD)	12.4 (15.5)	9.3 (13.6)	17.4 (16.8)
ADI, mean (SD)	37.8 (17.9)	28.1 (11.5)	53.1 (15.3)

NSAIDs: Non-steroidal antiinflammary drugs

ACN, advanced colorectal neoplasia; ADI, Area Deprivation Index; BMI, body mass index; CRC, colorectal cancer; NSAIDs, non-steroidal antiinflammatory drugs.

The univariate associations between explanatory variables and ACN are presented in online supplemental table 2. The prevalence of ACN is 7.1% in the entire population, 6.3% in the EAs and 8.4% in the AAs. The risk factors showing significant univariate association with ACN vary between EAs and AAs.

### Race-specific ACN prediction models without ADI

Three prediction models were developed, respectively, for the entire population, the EAs and the AAs using our model fitting and variable selection algorithm (table 2). For the entire population, race is forced to be included in the prediction model although it was not selected by variable selection algorithm. The prediction model for EAs (model 2) and the model for AAs (model 3) have only two predictors in common. The model for the whole population (model 1) is similar as model 2, with one more predictor. The Nagelkerke’s pseudo R^2^ is 4.9% for model 1, 5.4% for model 2 and 3.7% for model 3.

**Table 2:** The multivariable estimates of predictors in the prediction models of advanced colorectal neoplasia, overall and by race

	Model 1		Model 2		Model 3	
	All		EAs		AAs	
	OR (95% CI)	*P* value	OR (95% CI)	*P* value	OR (95% CI)	*P* value
Race (AA vs EA)	1.08 (0.75 to 1.54)	0.680				
Sex (male vs female)	1.41 (1.00 to 1.99)	0.050	1.43 (0.91 to 2.27)	0.124		
Age (year)	1.03 (1.01 to 1.06)	0.006	1.03 (1.00 to 1.06)	0.055	1.03 (1.00 to 1.07)	0.044
BMI (kg/m^2^)	1.03 (1.00 to 1.05)	0.040	1.04 (1.00 to 1.08)	0.027		
Family history of CRC in first degree relatives	1.69 (1.09 to 2.64)	0.026	1.83 (1.01 to 3.31)	0.046		
Years of smoking	1.01 (1.00 to 1.02)	0.050	1.01 (1.00 to 1.03)	0.152	1.01 (1.00 to 1.02)	0.178
Diabetes	1.49 (1.00 to 2.23)	0.058			1.76 (1.06 to 2.91)	0.029
Calcium	0.59 (0.35 to 1.00)	0.039	0.44 (0.22 to 0.87)	0.018		
Red meat per week (frequency)					1.04 (0.98 to 1.10)	0.178

Each model only includes the final predictors that are selected by the bootstrap selection procedures, and estimates are evaluated in the corresponding population.

AA, African American; BMI, body mass index; CRC, colorectal cancer; EA, European American.

As shown in table 3, model 1 demonstrated good discrimination (C-statistic=0.653 (95% CI 0.607 to 0.698) in the combined population but poor calibration accuracy across deciles of predicted risk (calibration *P*=0.053). It had good prediction accuracy in the EAs (C-statistic of 0.653 (95% CI 0.591 to 0.716), calibration *P*=0.735), but poor calibration accuracy in the AAs (calibration *P*=0.011).

**Table 3:** Prediction performance of ACN risk prediction models

	Model 1		Model 2	Model 3
Population	Overall		European Americans	African Americans
C-statistic (95% CI)	0.653 (0.607 to 0.698)		0.655 (0.594 to 0.717)	0.637 (0.572 to 0.702)
*P*_Calibration_*	0.053		0.663	0.810
Apply to sub population	European Americans	African Americans		
C-statistic (95% CI)	0.653 (0.591 to 0.716)	0.635 (0.566 to 0.703)		
*P*_Calibration_*	0.735	0.011		

*By Hosmer-Lemeshow test for agreement between observed and predicted risk of ACN across deciles of predicted risk probabilities.

ACN, advanced colorectal neoplasia.

Compared with model 1, the race-specific prediction models (model 2 and model 3) had fewer predictors but better prediction accuracy than model 1 in their corresponding race groups. Model 2 predicted well in the EAs with C-statistic=0.655 (95% CI 0.594 to 0.717) and calibration *P*=0.663. Similarly, prediction accuracy of model 3 in AAs is C-statistic=0.637 (95% CI 0.572 to 0.702) and calibration *P*=0.810.

### Adding ADI in ACN prediction

The ADI accounted for 60.2% of the overall variability of the 17 nSES variables, and the poverty rate (proportion of population below 150% of the federal poverty threshold) contributed the most to ADI (online supplemental table 3).

In univariate association analysis, ADI was significantly associated with ACN in the EAs (*P*=0.041, online supplemental table 2) but not in the AAs. Similarly, for EAs, the ACN prevalence has a significant trend of increase with the ADI quantiles (*P*_Trend_=0.026, online supplemental table 4), but for AAs, the trend is not significant (*P*=0.297).

For the whole (EA+AA) population, ADI was not a significant predictor after adding it to the ACN risk prediction model (*P*=0.209, table 4). In stratified analyses, however, ADI is a significant predictor of ACN for EAs after being included in the prediction model (*P*=0.029). In contrast, for AAs, ADI was not a significant predictor of ACN after its inclusion in the model (*P*=0.487).

**Table 4:** The multivariable estimates of ACN prediction with addition of ADI in mixed models*

	Model 1’		Model 2’		Model 3’	
	EA+AA		EA		AA	
	OR (95% CI)	*P* value†	OR (95% CI)	P value†	OR (95% CI)	*P* value†
Predicted risk from model i ‡	2.95 (2.01 to 4.32)	1.58×10^−7^	3.84 (2.29 to 6.47)	9.44×10^−7^	2.35 (1.27 to 4.34)	0.009
ADI§	1.07 (0.97 to 1.18)	0.209	1.24 (1.03 to 1.50)	0.029	1.07 (0.89 to 1.28)	0.487

*Model i': ACN~predicted risk from model i+ADI+(1|census tract), i=1, 2, 3.

†Statistical significance was evaluated by likelihood ratio test in the mixed effect logistic regression model.

‡The predicted risk (absolute risk in probability, range 0–1) by the corresponding model without ADI (model i in table 3, i=1, 2, 3), OR is for unit predicted risk of 0.1.

§OR for ADI score of 10 (ADI score range is 0–100).

AA, African American; ACN, advanced colorectal neoplasia; ADI, Area Deprivation Index; EA, European American.

**Figure 1:**
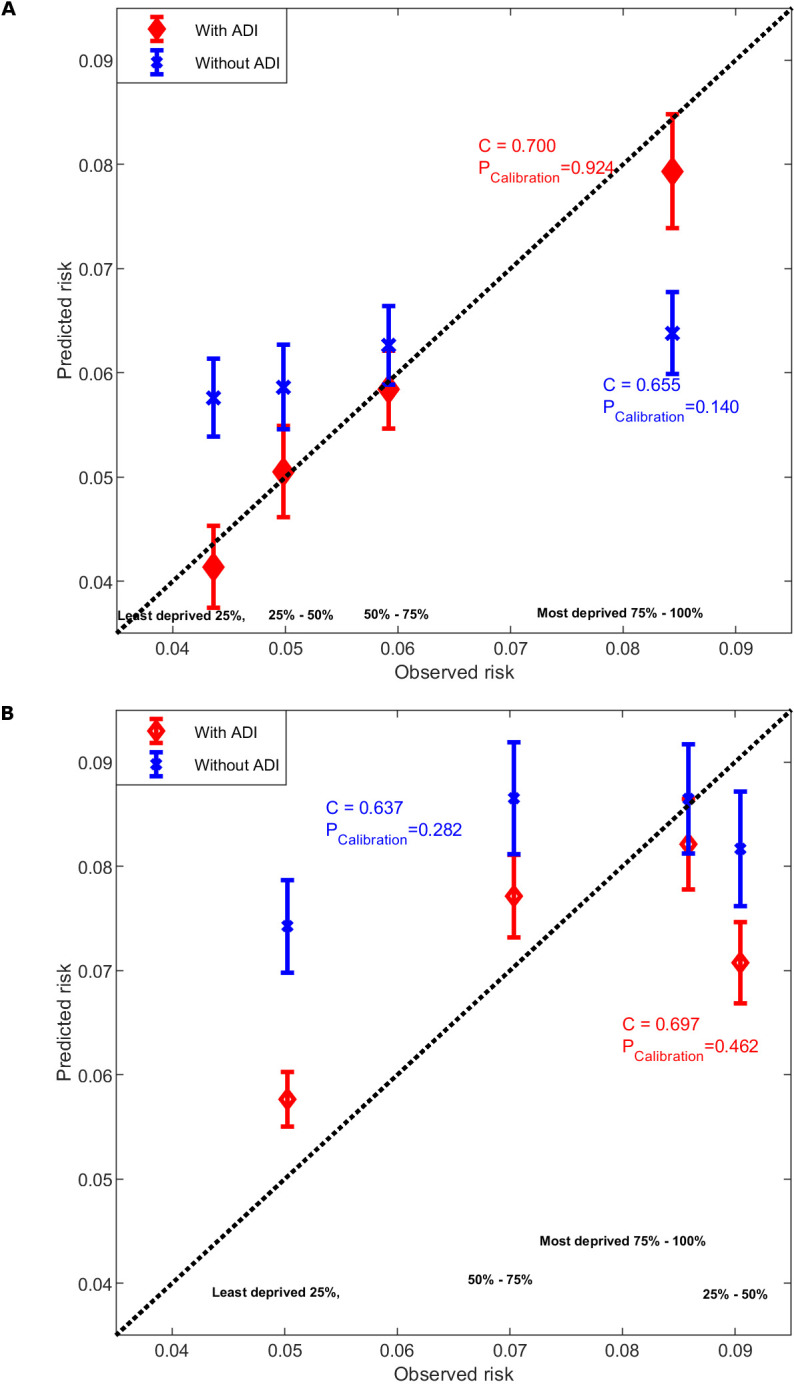
The predicted ACN risk versus observed ACN risks across four quantiles of Area Deprivation Index (ADI). (A) For European Americans; predicted risk before (blue, by model 2) and after adding ADI (red, by model 2’) in the prediction. (B) For African Americans; predicted risk before (blue, by model 3) and after adding ADI (red, by model 3’) in the prediction. The x-axis is the observed ACN risk, that is, the prevalence of ACN in the four ADI subgroups, y-axis is the mean predicted ACN risks. The diagonal line is the line when predicted risk equals observed risk. *P* values are the calibration accuracy by Hosmer-Lemeshow test for agreement between observed and predicted risk of ACN across quantiles of ADI. ACN, advanced colorectal neoplasia.

As shown in figure 1, for EAs, the prediction without ADI did not calibrate well across the area deprivation subgroups. It overestimated average ACN risk for those from the least deprived neighbourhoods and underestimated the average ACN risk for those from the most deprived neighbourhoods. The prediction model with ADI (by model 2’) had an improved calibration accuracy across the area deprivation groups compared with the prediction without ADI (by model 2) (*P*_Calibration_=0.924 for the prediction with ADI vs *P*_Calibration_=0.140 for the prediction without ADI), and it also had a better discrimination accuracy (C-statistic=0.700 vs C-statistic=0.655). For AAs, the ACN prediction with ADI had limited improvement in the calibration across quantiles of ADI (*P*_Calibration_=0.462 for the prediction with ADI vs *P*_Calibration_=0.282 for the prediction without ADI).

For EAs, ADI accounted for 38.7% of census tract-level variation of ACN risks by comparing the random variances of the null model (ACN~predicted risk from model 2+(1|census tract)) and the random variance of the full model (ACN~predicted risk from model 2+ADI+(1|census tract)). For AAs, ADI only accounted for 10.6% of census tract-level variation of ACN risks.

## Discussion

We performed an extensive study of the impact of nSES on ACN risk prediction. Our study indicated that ADI was significantly associated with ACN in EAs, and adding ADI to the ACN prediction model for EAs effectively improved the calibration accuracy across area deprivation subgroups.

nSES reflects area or geographical disparity in healthcare availability, affordability, acceptability and accessibility, and it may influence cancer outcomes of the residents.^37^ Our study is motivated by previous studies that found association between neighbourhood socioeconomic disadvantage and CRC risk^3 9^ and a study using nSES to improve cardiovascular risk prediction.^12^ We found that without adding ADI, the prediction model for EAs showed overprediction of ACN risk for those living in affluent neighbourhoods, and underprediction of risk for those living in disadvantaged neighbourhoods. Such discrepancy of calibration across area deprivation groups was corrected by adding ADI to the risk prediction model. Unlike the EA patients, we found that ACN prevalence in the AA patients does not exhibit a significant trend of increase with ADI. Additionally, the association of ADI with can in AAs was non-significant, regardless of whether the random effect of the census tract was considered in our analyses. Consequently, including ADI in the prediction model had limited improvement in the calibration accuracy across area deprivation groups in AAs. This limitation may stem from the non-linear relationship between ADI and the observed ACN risk among AAs. Specifically, the risk of ACN appears higher for patients in the second quantile of ADI compared with those in the other three quantiles (figure 1B). Thus, the linearity assumption of the risk prediction model using logistic regression may not adequately account for the complexity of this relationship. In addition, the AA participants in this study are mainly from much more disadvantaged neighbourhoods in Cleveland than the EA participants (table 1, mean ADI=53.11 for AAs vs mean ADI=28.09 for EAs, *P*=2.37×10^−240^). Ladabaum *et al* found a similar racial difference in the association between nSES and CRC incidence, with the association being significant among non-Hispanic whites, but considerably smaller among Asian/Pacific Islanders.^38^ Moreover, ADI can only explain a small portion of census tract-level variation in ACN risk in AAs, indicating the presence of other environmental risk factors not captured by nSES.

The finding of nSES disparity in ACN risk prediction is intriguing. nSES reflects the social determinants of health, the conditions to which people are exposed during their life course that are shaped by the distribution of money, power and resources.^39^ nSES can significantly impact health outcomes by altering the conditions that individuals or populations face. It affects their exposure to risk factors, their access to healthful behaviours and healthcare resources, and their ability to manage their health effectively.^40^ The observed disparity of nSES in ACN risks may in part be attributable to the higher prevalence of adverse health behaviours in low-SES populations.^9^

We found EAs and AAs had differences in ACN risk and risk factors, and this racial disparity in ACN led to our development of race-specific ACN prediction models. In our study, AAs have higher prevalence of ACN than EAs, which is consistent with other studies that found racial/ethnic disparities in the risk of ACN or colorectal adenoma.^2 41^ In univariable association analysis, age, years of smoking and diabetes show weak associations in both EAs and AAs (*P*<0.10), although none of them are significantly associated with ACN risk in both racial populations. However, calcium and BMI were only significantly associated with ACN in EAs. Other studies also found such racial differences, for example, two studies found BMI and waist circumference were only significantly associated with colon adenoma in whites,^42 43^ which is consistent with our results. Such differences in association might reflect the racial difference in underlying ACN exposures and their impact on outcome.

The prediction model fitted by the entire population (model 1) predicted well in the EAs but showed poorer performance in the AA population. Conversely, race-specific prediction models developed for EAs and AAs, with fewer predictors, exhibited better prediction accuracy within their respective racial/ethnic groups compared with the overall model. Notably, there is currently no reported ACN prediction model specifically tailored for AAs. The model reported by Schroy *et al* 22 was developed using a diverse population, including mainly whites and blacks, and some Hispanics. Similar to our model 1, its prediction accuracy in blacks was lower than in whites. To bridge this gap, we developed the ACN prediction model tailored specifically for the AA population (model 3), which demonstrated the highest prediction accuracy for AAs among the three models we developed.

It is difficult to have one ACN prediction model fitting for multiple races/ethnicities. Model 2 for EAs did not predict well in the AAs (online supplemental table 5, C-statistic=0.608; calibration *P*=0.018), and model 3 for AAs did not predict well in EAs (C-statistic=0.586; calibration *P*=0.369). Although a number of ACN risk prediction models have been developed based on populations in Western and Asian countries,^19–24^ and a recent study found that a CRC prediction model may also have the potential to predict ACN risk,^44^ we found that four published ACN prediction models that were developed from various populations (EAs, multiple races or Asian)^20 22–24^ had lower discrimination and very poor calibration performance in our population (C-statistics <0.61 and calibration *P* values ≤0.01 for our EA, AA and the entire population, online supplemental table 6), which may further indicate the limitation of the current ACN prediction models and the complexity of generalising ACN prediction models to other races or populations. The limited generalisability of ACN prediction to external populations of the same or different races was also reported by other studies.^45^ Such limitation might result from the differences in population exposures such as socioeconomic factors and lifestyle risk factors, or differences in tumour molecular mechanisms or sample ascertainment criteria. Alternatively, this limited generalisability could also be due to model overfitting.

In order to obtain robust and stable prediction models with our relatively limited sample size, we performed 1000 bootstrap samplings for model fitting and backward selection, and obtained 1000 best-fitting models that only predictors with multivariable *P* values <0.1 were kept in each of them. The predictors that have been stably selected, that is, most frequently appeared in the 1000 best-fitting models, were selected as the final predictors. Therefore, although a predictor may have a multivariable *P* value >0.1 estimated in the original dataset (as shown in table 2), in each of the sampled datasets, its multivariable *P* value was <0.1. Such bootstrap sampling along with an automated selection procedure has been used to obtain stable models, and it has been demonstrated that the identified variables are important predictors.^29^ It is well known that traditional stepwise model selection methods, such as backward elimination, forward selection and bidirectional elimination, have disadvantages, especially when the sample size is small. They have limited power to select true variables, are likely to include noise variables in the final model, may lead to biased coefficients and overfitting, and consequently, they can produce unstable models.^46^ Bootstrapping variable selection methods have been shown to effectively mitigate model instability.^29 47^ In our study, we used a significance level of *P*=0.10 as the stopping cut-off for variable selection. This threshold is between 0.05 and *P* value of 0.157,^48^ which corresponds to the popular AIC criterion^49^ to avoid missing true predictors and to produce better predictive performance,^50^ although it may result in a less parsimonious model.

### Limitations

Our study sample size is relatively limited and does not allow for split-sample validation. Independent validation of our ACN prediction models is needed.

### Strengths

Our study represents the first effort to incorporate nSES into ACN risk prediction. The results from this study strongly suggest that failure to take into account neighbourhood deprivation in ACN risk prediction may lead to biased risk assessment and further worsen health disparities for those living in disadvantaged communities. This insight may help to guide future research and potentially influence targeted prevention policies for colorectal neoplasia.

## Conclusion

Our results suggested that nSES is an important predictor of ACN. Inclusion of ADI in ACN risk prediction for EAs improved prediction accuracy for both those from disadvantaged neighbourhood and those from affluent neighbourhood. In addition, there is no one-fit-all ACN prediction models for all races, race-specific ACN prediction models are needed for precision ACN prediction across diverse racial populations.

## Data Availability

Data are available on reasonable request.
