# Supplementary material for: Risk prediction of advanced colorectal neoplasia varies by race and neighbourhood socioeconomic status
Source: Fam Med Community Health. 2024 May 30;12(Suppl 2):e002892. doi: 10.1136/fmch-2024-002892 (PMC11141178; doi:10.1136/fmch-2024-002892)
Supplement: Supplementary data [file fmch-2024-002892supp001.pdf]

Risk prediction of advanced colorectal neoplasia varies by race and neighborhood socioeconomic status

Supplementary materials

Evaluating prediction performance of published ACN prediction models in our population

Predictors of four existing ACN prediction models were measured in our data, so we evaluated their prediction performance in our population to study their generalizability to other population. The prediction model by Imperiale et al[1] was developed from a cohort of European Americans in Indiana and Northeast Ohio, and the prediction model by Schroy et al[2] was developed from a diverse population of a mixture of whites, blacks, Asians, and Hispanics in Boston area. The Asia-Pacific Colorectal Screening (APCS) model was developed by Yeoh et al with an Asian population from 11 Asian cities,[3] and a modified APCS model by Sung et al included an additional predictor BMI.[4] These prediction models were all fitted by multivariable logistic regression. In evaluating the model in our data, we fixed the coefficients of the predictors of the logistic model to the reported estimates, and then re-estimated the intercept, which denotes the baseline risk, of the logistic model. The risk of ACN for each subjects in our data was predicted using the model, and the model's prediction performance (calibration and discrimination accuracy) in our population was evaluated.

Results of Other ACN models' prediction performance in our population

The above results suggested that ACN risk factors and prediction models may be race/ethnic specific and hard to generalize to other populations. In order to validate if other published ACN prediction models are population specific, we studied the prediction accuracy of four existing ACN models in our population. The predictors of the four models were measure in our study and were extracted from our data. The predictors in the model by Imperiale et al[1] are age, sex, family history of colorectal cancer in first degree relatives, waist circumference, and pack year of smoking. The predictors in the model by Schroy et al[2] are age, race by sex interaction, smoking years, drinks of alcohol, and height. The predictors in the APCS model[3] are age, sex, family history of colorectal cancer in first degree relatives, and smoking history, and BMI was the additional predictor in the modified APCS model by Sung et al[4] 2018. The discrimination accuracy (C-statistics) of these models in our entire population, in the EAs, and in the AAs are all ≤ 0.61 (Supplementary Table 6), and their calibration performance of agreement between observed and predicted outcomes were not good in either our EA, AA, or the entire population (calibration P values ≤ 0.01).

Supplementary Table 1 The distribution (Mean (SD)) of neighborhood SES variables in the study population and their univariable association with advanced colorectal neoplasia

	All European- and African-Americans				European Americans				African Americans			
	Unaffected	Affected	P[a]	OR (95% CI)	Unaffected	Affected	P[a]	OR (95% CI)	Unaffected	Affected	P[a]	OR (95% CI)
Median family income, x $1k	75.89 (38.41)	68.89 (38.70)	0.031	0.995 (0.9905,0.9995)	93.99 (32.67)	87.35 (33.44)	0.074	0.9933 (0.9863,1.0004)	46.39 (27.16)	44.64 (31.12)	0.657	0.9975 (0.9879,1.0073)
Income disparity	2.67 (1.71)	3.06 (1.75)	0.008	1.137 (1.036,1.247)	1.72 (1.09)	1.91 (1.19)	0.166	1.158 (0.957,1.402)	4.13 (1.45)	4.48 (1.20)	0.023	1.196 (1,1.431)
Families below poverty level %	14.13 (15.63)	16.42 (15.66)	0.076	1.0086 (0.9991,1.0183)	5.97 (7.13)	7.26 (8.58)	0.171	1.021 (0.996,1.047)	26.88 (16.73)	27.61 (15.09)	0.697	1.0026 (0.9884,1.0171)
Population below 150% of the poverty threshold %	26.03 (20.99)	30.55 (21.83)	0.012	1.0096 (1.0024,1.0169)	14.15 (11.60)	16.72 (13.24)	0.079	1.016 (1,1.032)	44.61 (18.78)	47.46 (18.05)	0.203	1.0083 (0.9952,1.0215)
Single-parent households with children aged < 18 %	11.51 (8.88)	11.87 (8.31)	0.594	1.0046 (0.9869,1.0225)	7.28 (4.85)	8.09 (5.38)	0.172	1.031 (0.991,1.072)	18.12 (9.69)	16.50 (8.93)	0.147	0.982 (0.956,1.008)
Households without a motor vehicle %	13.53 (15.00)	16.75 (17.72)	0.026	1.012 (1.003,1.022)	5.96 (6.81)	5.87 (7.07)	0.915	0.9982 (0.9667,1.0308)	25.35 (16.57)	30.03 (17.79)	0.034	1.016 (1.002,1.03)
Households without a telephone %	2.42 (2.72)	2.57 (2.54)	0.469	1.02 (0.964,1.079)	1.73 (2.04)	1.57 (1.32)	0.296	0.956 (0.846,1.08)	3.49 (3.25)	3.79 (3.09)	0.438	1.027 (0.958,1.102)
Occupied housing units without complete plumbing %	0.35 (0.82)	0.32 (0.76)	0.633	0.953 (0.771,1.176)	0.23 (0.56)	0.21 (0.45)	0.674	0.927 (0.607,1.417)	0.53 (1.08)	0.45 (1.01)	0.522	0.926 (0.723,1.187)
Owner-occupied housing units %	63.61 (24.40)	59.83 (26.10)	0.077	0.9939 (0.9875,1.0003)	75.66 (18.38)	75.44 (18.58)	0.915	0.9994 (0.9878,1.0111)	44.78 (20.40)	40.74 (20.78)	0.117	0.9902 (0.9784,1.0022)
Households with more than 1 person per room %	0.91 (1.45)	0.93 (1.45)	0.813	1.013 (0.909,1.129)	0.65 (1.09)	0.49 (0.93)	0.109	0.832 (0.644,1.074)	1.30 (1.81)	1.48 (1.76)	0.403	1.054 (0.93,1.194)
Median monthly mortgage, x $100	14.09 (4.65)	13.31 (4.64)	0.043	0.962 (0.926,0.999)	15.87 (4.36)	15.07 (4.36)	0.100	0.955 (0.905,1.008)	11.26 (3.57)	11.09 (4.03)	0.732	0.986 (0.919,1.059)
Median gross rent, x $100	9.21 (3.78)	8.20 (2.86)	0.00007	0.913 (0.863,0.965)	10.54 (3.98)	9.44 (2.90)	0.002	0.911 (0.845,0.983)	7.23 (2.34)	6.79 (2.07)	0.089	0.914 (0.817,1.023)
Median home value, x $1k	153.23 (89.22)	137.93 (88.25)	0.039	0.9979 (0.996,0.9999)	193.13 (80.48)	180.29 (78.66)	0.142	0.9979 (0.9951,1.0007)	87.67 (59.04)	81.46 (66.20)	0.463	0.998 (0.9933,1.0028)
Employed persons aged >=16 in white-collar occupations %	63.76 (15.95)	60.40 (15.66)	0.010	0.987 (0.978,0.997)	70.80 (11.99)	66.44 (13.18)	0.003	0.973 (0.957,0.989)	52.74 (15.11)	53.02 (15.35)	0.884	1.0012 (0.9854,1.0172)
Civilian labor force population aged> =16 unemployed %	9.91 (9.22)	11.30 (9.37)	0.072	1.015 (0.999,1.031)	5.05 (3.56)	5.68 (3.66)	0.120	1.043 (0.991,1.097)	17.51 (10.17)	18.16 (9.66)	0.584	1.0063 (0.983,1.0303)
Population aged> =25 with < 9 years of education %	2.47 (2.44)	2.79 (2.71)	0.148	1.048 (0.989,1.111)	1.75 (1.91)	1.95 (1.99)	0.376	1.047 (0.949,1.155)	3.59 (2.74)	3.82 (3.10)	0.544	1.029 (0.947,1.117)
Population aged >=25 with at least a high school diploma %	89.65 (8.84)	87.87 (9.50)	0.023	0.98 (0.964,0.996)	93.88 (5.12)	93.34 (4.82)	0.318	0.982 (0.945,1.02)	83.03 (9.37)	81.19 (9.56)	0.120	0.98 (0.955,1.005)

[a] By t test assuming unequal variances

BMJ Publishing Group Limited (BMJ) disclaims all liability and responsibility arising from any reliance placed on this supplemental material which has been supplied by the author(s)

Supplementary Table 2 The univariable associations between each characteristic with advanced colorectal neoplasia

	European Americans + African Americans				European Americans				African Americans			
	Unaffected	Affected	OR[a] (95% CI)	P[a]	Unaffected	Affected	OR[a] (95% CI)	P[a]	Unaffected	Affected	OR[a] (95% CI)	P[a]
Number of individuals	2222	171			1365	92			857	79		
Sex, n (%)												
Female	1369 (61.6)	90 (52.6)	Ref		786 (57.6)	40 (43.5)	Ref		583 (68.0)	50 (63.3)	Ref	
Male	853 (38.4)	81 (47.4)	1.44 (1.06,1.97)	0.022	579 (42.4)	52 (56.5)	1.76 (1.15,2.70)	0.009	274 (32.0)	29 (36.7)	1.23 (0.76,1.99)	0.394
Race, n (%)												
European American	1365 (61.4)	92 (53.8)	Ref									
African American	857 (38.6)	79 (46.2)	1.37 (1.00,1.87)	0.051								
Age, years, mean (SD)	57.7 (7.1)	59.4 (7.6)	1.03 (1.01,1.05)	0.003	57.7 (7.0)	59.1 (7.5)	1.03 (1.00,1.06)	0.060	57.7 (7.3)	59.8 (7.7)	1.04 (1.01,1.07)	0.021
BMI, kg/m2, mean (SD)	29.5 (6.9)	31.2 (7.5)	1.03 (1.01,1.05)	0.005	28.2 (5.9)	30.0 (6.0)	1.05 (1.02,1.08)	0.006	31.8 (7.8)	32.5 (8.8)	1.01 (0.98,1.04)	0.441
Waist circumference, cm, mean (SD)	38.8 (6.5)	40.2 (7.2)	1.03 (1.01,1.06)	0.011	37.8 (6.2)	39.3 (6.8)	1.04 (1.00,1.07)	0.036	40.5 (6.6)	41.3 (7.6)	1.02 (0.98,1.05)	0.385
Waist Hip Ratio, mean (SD)	9.1 (0.9)	9.3 (1.0)	1.19 (1.01,1.41)	0.044	9.0 (0.9)	9.1 (1.0)	1.14 (0.89,1.45)	0.306	9.4 (0.9)	9.5 (1.0)	1.18 (0.92,1.51)	0.198
Height, cm, mean (SD)	169.1 (9.9)	170.1 (10.0)	1.01 (0.99,1.03)	0.232	170.1 (9.6)	171.8 (10.1)	1.02 (1.00,1.04)	0.106	167.5 (10.3)	168.0 (9.4)	1.01 (0.98,1.03)	0.643
Family history of CRC in all relatives (%)	479 (22.3)	44 (27.7)	1.33 (0.93,1.91)	0.128	321 (24.1)	25 (28.7)	1.27 (0.79,2.06)	0.336	158 (19.4)	19 (26.4)	1.49 (0.86,2.58)	0.171
Family history of CRC in first degree relatives (%)	228 (10.5)	27 (16.4)	1.68 (1.09,2.59)	0.027	139 (10.3)	15 (16.9)	1.77 (0.99,3.16)	0.069	89 (10.7)	12 (15.8)	1.56 (0.81,3.00)	0.202
Diabetes (%)	315 (14.5)	41 (25.2)	1.98 (1.36,2.87)	<0.001	118 (8.8)	13 (14.8)	1.80 (0.97,3.34)	0.078	197 (23.9)	28 (37.3)	1.90 (1.16,3.11)	0.014
Physical activities, MET, mean (SD)	3.3 (2.6)	3.3 (2.6)	1.00 (0.94,1.06)	0.968	3.7 (2.8)	3.7 (2.7)	0.99 (0.92,1.07)	0.848	2.6 (2.3)	2.868 (2.4)	1.06 (0.95,1.17)	0.292
Red Meat per week, Frequencies, mean (SD)	3.0 (3.2)	3.4 (3.5)	1.03 (0.99,1.08)	0.136	2.9 (2.8)	3.0 (2.3)	1.02 (0.95,1.09)	0.670	3.3 (3.8)	3.9 (4.5)	1.04 (0.99,1.10)	0.162
Alcohol per week, Frequencies, mean (SD)	3.0 (9.3)	3.1 (6.0)	1.00 (0.98,1.02)	0.892	3.0 (5.6)	3.9 (6.3)	1.02 (0.99,1.05)	0.182	3.1 (13.4)	2.247 (5.5)	0.99 (0.96,1.02)	0.519
NSAID (%)	708 (32.5)	64 (38.8)	1.32 (0.95,1.82)	0.103	477 (35.3)	32 (36.0)	1.03 (0.66,1.61)	0.902	231 (28.0)	32 (42.1)	1.87 (1.16,3.03)	0.012
Aspirin (%)	577 (26.5)	59 (35.8)	1.54 (1.11,2.15)	0.012	380 (28.1)	28 (31.5)	1.17 (0.74,1.86)	0.503	197 (23.9)	31 (40.8)	2.20 (1.36,3.57)	0.002
Ibuprophen (%)	216 (10.14)	11 (6.88)	0.65 (0.35,1.23)	0.163	157 (11.90)	10 (11.77)	0.99 (0.5,1.95)	0.970	59 (7.27)	1 (1.33)	0.17 (0.02,1.26)	0.020
Calcium (%)	451 (20.7)	20 (12.1)	0.53 (0.33,0.85)	0.005	360 (26.6)	11 (12.4)	0.39 (0.20,0.74)	0.001	91 (11.0)	9 (11.8)	1.08 (0.52,2.25)	0.828
Years of smoking, mean (SD)	12.1 (15.3)	16.3 (17.1)	1.02 (1.01,1.03)	0.001	9.1 (13.5)	12.4 (16.0)	1.68 (1.05,2.70)	0.033	17.1 (16.8)	20.9 (17.4)	1.51 (0.94,2.44)	0.088
ADI, mean (SD)	37.58 (17.92)	41.52 (17.46)	1.13 (1.03,1.23)	0.012	27.92 (11.46)	30.74 (12.19)	1.22 (1.01,1.46)	0.041	52.95 (15.44)	55.05 (13.11)	1.10 (0.92,1.30)	0.292

Abbreviations: ADI: area deprivation index; BMI: body mass index; CI: confidence interval; CRC: colorectal cancer; OR: odds ratio; SD: standard deviation.

[a] P values by likelihood ratio test

Supplementary Table 3 The loadings of Area Deprivation Index which represent correlation between neighborhood socioeconomic variables and Area Deprivation Index

Neighborhood SES variables	Loadings of Area Deprivation Index
Median family income	-0.286
Income disparity	0.287
Families below poverty level	0.284
Population below 150% of the poverty threshold	0.299
Single-parent households with children aged < 18	0.243
Households without a motor vehicle	0.259
Households without a telephone	0.145
Occupied housing units without complete plumbing	0
Owner-occupied housing units	-0.246
Households with more than 1 person per room	0.134
Median monthly mortgage	-0.252
Median gross rent	-0.210
Median home value	-0.264
Employed persons aged >=16 in white-collar occupations	-0.261
Civilian labor force population aged> =16 unemployed	0.270
Population aged> =25 with < 9 years of education	0.200
Population aged >=25 with at least a high school diploma	-0.276

Supplementary Table 4 Prevalence of advanced colon neoplasia by NDI levels

	Groups of patients according to quantiles of NDI				
	1st quantile	2nd quantile	3rd quantile	4th quantile	P_{Trend}
EA+AA	518	518	518	519	
	0.042	0.058	0.083	0.077	0.008
	(n = 321)	(n = 321)	(n = 321)	(n = 320)	
European Americans	0.044	0.050	0.059	0.084	0.026
	199	199	199	198	
African Americans	0.050	0.090	0.070	0.086	0.297

Supplementary Table 5 Prediction accuracy of race-specific ACN models in the same and other races

	Model 1		Model 2	Model 3
Population	Overall		European Americans	African Americans
C-statistic (95% CI)	0.653 (0.607, 0.698)		0.655 (0.594, 0.717)	0.637 (0.572, 0.702)
$P_{Calibration}$[a]	0.053		0.663	0.810
Apply to other population	European Americans	African Americans	African Americans	European Americans
C-statistic (95% CI)	0.653 (0.591, 0.716)	0.635 (0.566, 0.703)	0.608 (0.539, 0.677)	0.586 (0.523, 0.649)
$P_{Calibration}$[a]	0.735	0.011	0.018	0.369

Supplementary Table 6 The prediction performance of published ACN prediction models in original population and in our population

		Imperiale's model[1]	Schroy's model[2]	APCS model[3]	Modified APCS model[4]
	Original population	EA	EA + AA + Hispanic	Asian	Chinese
Reported prediction[a]	C-statistic (95% CI)	0.720	0.690 (0.660, 0.720)	0.660 (0.620, 0.700)	0.649 (0.608, 0.691)
	$P_{Calibration}$	0.420	0.730 - 0.930	0.290	0.571
Prediction in our population					
All EA and AA	C-statistic (95% CI)	0.601 (0.554, 0.648)	0.584 (0.534, 0.635)	0.596 (0.553, 0.640)	0.563 (0.515, 0.611)
	$P_{Calibration}$	7.103×10^{-6}	5.075×10^{-8}	7.423×10^{-5}	1.190×10^{-4}
EA	C-statistic (95% CI)	0.607 (0.542, 0.673)	0.590 (0.518, 0.662)	0.602 (0.541, 0.663)	0.548 (0.481, 0.615)
	$P_{Calibration}$	8.206×10^{-3}	3.681×10^{-4}	4.185×10^{-5}	8.796×10^{-5}
AA	C-statistic (95% CI)	0.578 (0.508, 0.648)	0.586 (0.514, 0.658)	0.589 (0.527, 0.651)	0.570 (0.502, 0.639)
	$P_{Calibration}$	3.588×10^{-3}	3.087×10^{-3}	1.575×10^{-3}	3.213×10^{-3}

[a] Adapted from references 1-4

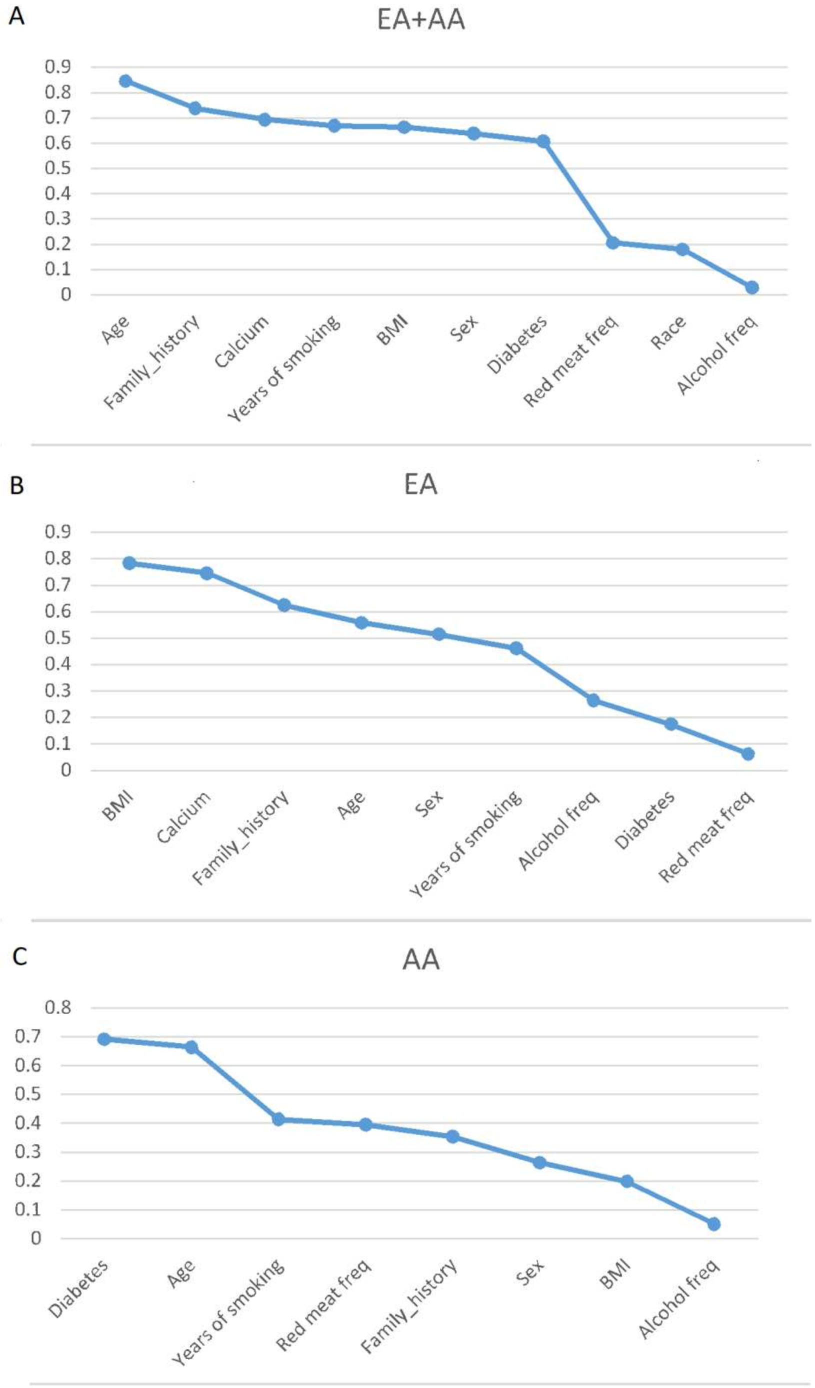

Supplementary Figure 1 The frequencies of the variables being selected by backward model selection under 1000 bootstrap samplings. X axis is the potential risk factors for the prediction model, Y axis is the frequency of being selected in the 1000 best fitting models by bootstrap sampling.

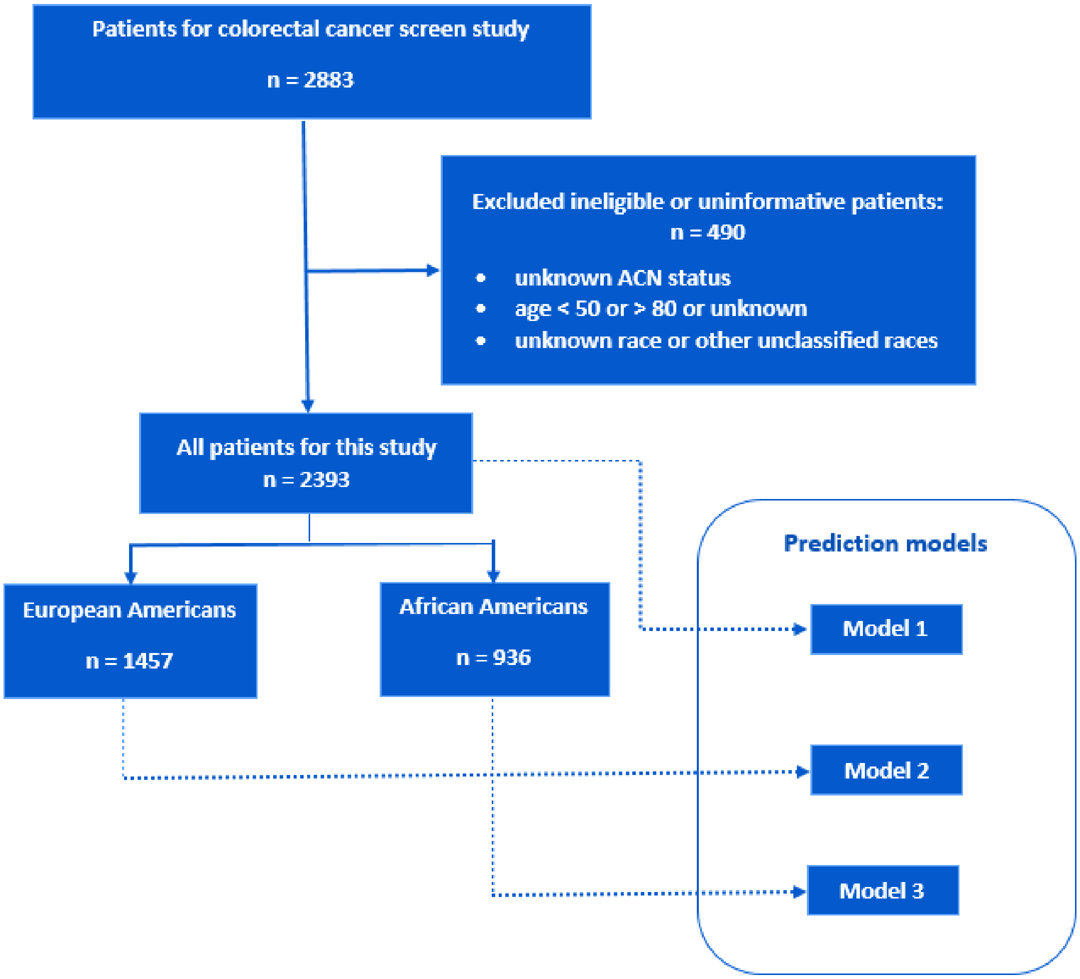

Supplementary Figure 2. The study flow chart

References

1. Imperiale TF, Monahan PO, Stump TE*, et al.* Derivation and Validation of a Scoring System to Stratify Risk for Advanced Colorectal Neoplasia in Asymptomatic Adults: A Cross-sectional Study. *Ann Intern Med* 2015;163(5):339-46. doi: 10.7326/m14-1720.
2. Schroy PC, 3rd, Wong JB, O'Brien MJ*, et al.* A Risk Prediction Index for Advanced Colorectal Neoplasia at Screening Colonoscopy. *Am J Gastroenterol* 2015;110(7):1062-71. doi: 10.1038/ajg.2015.146.
3. Yeoh KG, Ho KY, Chiu HM*, et al.* The Asia-Pacific Colorectal Screening score: a validated tool that stratifies risk for colorectal advanced neoplasia in asymptomatic Asian subjects. *Gut* 2011;60(9):1236-41. doi: 10.1136/gut.2010.221168.
4. Sung JJY, Wong MCS, Lam TYT*, et al.* A modified colorectal screening score for prediction of advanced neoplasia: A prospective study of 5744 subjects. *Journal of gastroenterology and hepatology* 2018;33(1):187-94. doi: 10.1111/jgh.13835.
